# Accuracy of Femoral Tunnel Placement between Anteromedial and Anterolateral Visualisation Portals in Anterior Cruciate Ligament Reconstruction - Outcomes of a CT based Cross-Sectional Study

**DOI:** 10.5704/MOJ.2307.002

**Published:** 2023-07

**Authors:** G Balaji, G Yadav, SA Patel, A Ramesh, S Nema, T Ramalingam

**Affiliations:** 1 Department of Orthopaedics, Jawaharlal Institute of Postgraduate Medical Education and Research (JIPMER), Pondicherry, India; 2 Department of Orthopaedics, Sarvajanik College of Physiotherapy Rampura, Surat, India

**Keywords:** tunnel placement, anatomic, anterior cruciate, portal

## Abstract

**Introduction:**

Anatomical femoral tunnel placement is critical for anterior cruciate ligament reconstruction (ACLR). Tunnel placement may vary with different surgical techniques. The aim of this study was to compare the accuracy of femoral tunnel placement between the Anteromedial (AM) and Anterolateral (AL) visualisation portals on post-operative CT scans among a cohort of ACLR patients.

**Materials and methods:**

This cross-sectional study was conducted from January 2018 to March 2020 after obtaining ethics clearance. Patients who went for arthroscopic ACLR in our institute were divided into an AM (group 1) and an AL (group 2) based on the visualisation portal for creating the femoral tunnel and a 3D CT scan was done. The femoral tunnel position was calculated in deep to shallow and high to low direction using the Bernard Hertel grid. Femoral tunnel angle was measured in the 2D coronal image. Statistical analysis was done with the data collected.

**Results:**

Fifty patients with an average age of 26.36 (18-55) years ±7.216 SD were enrolled in the study. In this study, the AM technique was significantly more accurate (p<0.01) than the AL technique in terms of femoral tunnel angle. Furthermore, the deep to the shallow position was significantly (p= 0.018) closer to normative values, as determined by the chi-square test. The chances of error in tunnel angle in femoral condyle are 2.6 times greater in the AL technique (minimal clinical difference).

**Conclusion:**

To conclude, in ACLR the anteromedial visualisation portal can facilitate accurate femoral tunnel placement compared to the anterolateral visualisation portal.

## Introduction

Injury to the ACL alters the normal knee kinematics resulting in knee arthritis. The primary aim of ACLR is to restore the normal knee kinematics. The reconstruction should be as anatomic as possible to avoid failure and arthritis. The success of ACL reconstruction depends on several factors, among which femoral tunnel positioning plays a crucial role in the outcome.

There is a variation in femoral tunnel placement among surgeons for several reasons, including their expertise, training, literature support etc. The placement of the femoral tunnel in arthroscopic ACLR is also dependent on the technique chosen for drilling the femoral tunnel. While transtibial drilling places the femoral tunnel anteriorly and vertically in the distal femur, the transportal technique directed independent femoral tunnel placement. Recently, there has been an emphasis on more anatomical femoral tunnel placement in ACLR which is closer to the native anterior cruciate ligament (ACL) footprint. Studies have shown that anatomic femoral tunnel placement can be obtained better by transportal technique compared to a transtibial technique^1-7^.

Transportal femoral tunnel can be created either by visualisation from an anterolateral (AL) portal and making the tunnel from anteromedial portal or by visualisation from an anteromedial (AM) portal and making the tunnel from the accessory anteromedial portal. We hypothesise higher accuracy with AM portal-based femoral tunnel creation in ACLR. CT scan is the standard and validated imaging technique to evaluate the tunnel placement since plain radiographs are unreliable, challenging and particularly difficult to assess femoral tunnel position in the lateral view. Hence, this study aimed to compare the accuracy of femoral tunnel placement between the AM and AL visualisation portals on post-operative CT scan among a cohort of ACLR patients.

## Materials and Methods

This cross-sectional study was conducted from January 2018 to March 2020 at a centre treating complex knee injuries. The institutional review board approved the study, and the study was completed in accordance with the Declaration of Helsinki. We searched the operation records to screen patients who underwent arthroscopic ACLR.

Patients with multi-ligamentous knee injuries, knee dislocations, and PCL injuries were excluded from the study. The cases were operated by two arthroscopic surgeons with more than 10 years of experience in sports medicine. We divided the cases into AM (group 1) and AL (group 2) based on the visualisation portal used for creating the femoral tunnel. In group 1 (AM), the femoral tunnel was drilled through the accessory anteromedial portal (AAM) by visualising from the anteromedial portal whereas in group 2 (AL), the femoral tunnel entry was made from the anteromedial portal by visualising through the anterolateral portal. The AL portal was placed high at the level of the inferior pole of patella adjacent to the lateral border of the patellar tendon. The AM portal was placed high at the same level as AL portal adjacent to the medial border of the patellar tendon. For the AAM portal, a 20G spinal needle was placed 1cm medial and lower to the AM portal and under direct vision by arthroscope from the AL portal it was confirmed to be above the anterior horn of medial meniscus as well as it had good access to the femoral tunnel entry site. In addition, care was also taken that the needle was not too close to the medial femoral condyle. Then, a portal was created with a 11-size blade.

For ACL reconstruction, a tourniquet was used for all the cases and the leg was in hanging position with a thigh holder. Different anatomical landmarks have been mentioned in the literature for anatomical femoral tunnel placement. In our institute, we routinely place the femoral tunnel slightly posterior to the centre of the native ACL footprint with the knee in 120° of flexion. In the absence of the native footprint, the femoral tunnel was usually placed inferior to the lateral intercondylar ridge and slightly posterior to the bifurcate ridge.

After obtaining informed consent, all these patients were contacted and included in this study. In the post-operative follow-up, a CT scan with 3D reconstruction was ordered for these patients.

**Fig 1:**
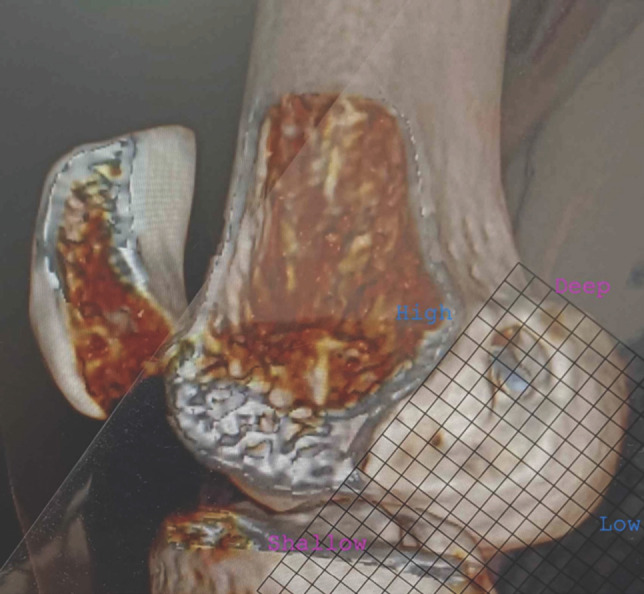
Figure showing the 3D reconstructed image of the medial side of the lateral femoral condyle with Bernard Hertel grid drawn in transparent sheet and placed over it to measure the femoral tunnel location in deep to shallow and high to low direction.

The three-dimensional sagittal image of the CT scan was formatted to subtract the medial condyle to assess the position of the femoral tunnel on a Bernard Hertel grid. The Bernard and Hertel’s grid were drawn on a plastic overhead transparent projector sheet of 10cm x 10cm dimension divided into multiple squares of 5mm x 5mm, and 1mm each of this grid was calibrated to 1%. The superior border of the grid was placed along the Blumensaat’s line. The Blumensaat’s line was drawn through the roof of the intercondylar notch placing the posterior border of the grid along the posterior femoral condyle. The femoral tunnel entry was recorded. The femoral tunnel position was calculated in deep to shallow and high to low direction (Fig. 1). Deep to shallow is defined as the femoral tunnel position, which is calculated parallel to the Blumensaat’s line as a percentage of the total sagittal diameter of the lateral femoral condyle and high to low is defined as femoral tunnel position calculated perpendicular to the Blumensaat’s line as a percentage to the maximum intercondylar notch height. A reference of 24-27% in the deep to shallow and 28-34% in the high to low direction was taken to estimate the femoral tunnel's position on the Bernard Hertel grid superimposed on the formatted sagittal image^8-10^. The values obtained from the operated cases were compared to the reference values described above.

**Fig 2:**
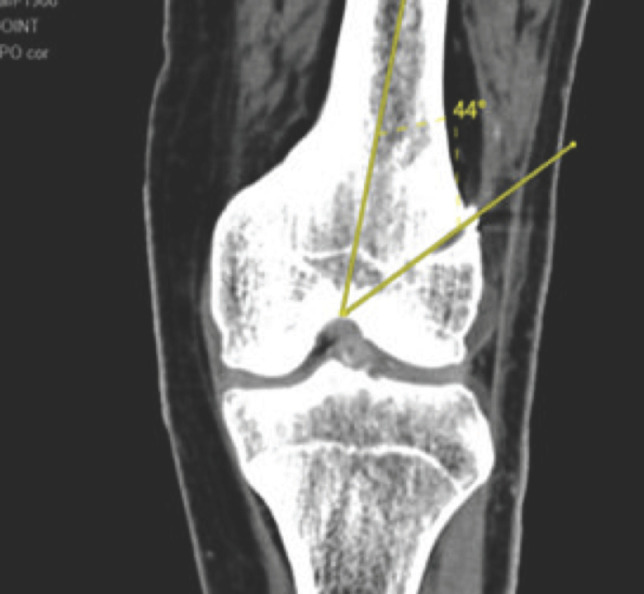
Coronal cut of the CT scan of knee joint showing measurement of the femoral tunnel angle.

Femoral tunnel angle was measured in the 2D coronal image by a line drawn through the tunnel with the shaft of the femur (Fig. 2). Normal reference for femoral tunnel angle was taken as greater than 35° and compared to those obtained from cases. Statistical analysis was performed with the means of statistical software SPSS for Windows, version 19 [SPSS, Chicago, IL].

## Results

Fifty patients with an average age of 26.36 (18-55) years ±7.216 SD were enrolled in the study. We had 46 (92%) males and 4 (8%) females among the enrolled patients. There were 21 (42%) left and 29 (58%) right ACLRs. 27 cases were in group 1, and 23 cases were in group 2.

The mean femoral tunnel position (deep-shallow, high-low, and in relation to Blumensaat’s line) and the femoral tunnel angle for both the techniques (Table I). Table II shows the percentage of near anatomic placement of the tunnel by both techniques.

**Table I:** Shows the mean femoral tunnel position and angle between the two groups

Femoral tunnel	Number	Group	Mean (in mm)	SD
Deep to shallow	27	1	26.07	1.21
	23	2	25.78	2.94
High to low	27	1	29.96	2.49
	23	2	30.65	1.87
Femoral tunnel angle	27	1	40.37	5.98
	23	2	35.00	11.27

**Table II:** Shows near anatomic placement data between the two groups

Femoral tunnel	Number	Group	Near anatomic placement	More than normal	Less than normal	Percentage
Deep to shallow	27	1	25	2	0	92.6
	23	2	16	1	6	69.5
High to low	27	1	25	1	1	92.6
	23	2	22	0	1	95.7
Femoral tunnel angle	27	1	22	0	5	81.5
	23	2	9	0	14	39.1

In this study, the AM technique was significantly more accurate (p<0.01) than the AL technique in terms of femoral tunnel angle. Furthermore, the deep to the shallow position was significantly (p=0.018) closer to normative values, as determined by the chi-square test. The chances of error in tunnel angle in femoral condyle are 2.6 times greater in the AL technique (minimal clinical difference).

## Discussion

Our study shows that the deep to shallow position of the femoral tunnel placement using the AM visualisation portal (AM technique) was significantly closer to normative values than that of the AL visualisation portal (AL technique). In addition, the AM technique was found to be significantly more accurate than the AL technique in terms of femoral tunnel angle. Fourteen patients who underwent surgery by AL technique had femoral tunnel angles that were outside the normal range.

In ACLR, we found that AM visualisation-based AAM femoral tunnel creation was more accurate than AL-based AM femoral tunnel placement. The observed difference between the two techniques could be attributed to the poor visualisation of the ACL footprint through the 30° arthroscope from the AL portal. Viewing through the AL portal gives an inaccurate estimate of the depth of the posterior border of the lateral femoral condyle. The main advantage of the AM visualisation, on the other hand, is a direct view of the ACL footprint and the medial surface of the lateral femoral condyle. It prevents the femoral tunnel from being created non-anatomically. The incomplete visualisation of the ACL footprint from the AL portal also contributes to incorrect border estimation. For accurate tunnel placement, a 70° arthroscope should be used to view the medial surface of the lateral condyle. However, the 70° arthroscope's infrequent use and skewed visualisation makes it difficult to use on a regular basis.

Though the instrument crowding was blamed as one of the determinants for use against viewing and working portals in the AM technique, we placed the anteromedial portal at a higher level and the accessory anteromedial portal at a lower and medial level which made it convenient to drill the ACL femoral tunnel.

Anatomical femoral tunnel placement is critical for anterior cruciate ligament reconstruction (ACLR). Multicentre ACL Revision Study (MARS) reported that 80% of ACLR failure was at least partially secondary to a mal-positioned femoral tunnel^11^. Even experienced surgeons find it difficult to place femoral tunnels anatomically due to variations in surgical techniques, bone anatomy and patient morphology. Tunnel placement techniques have evolved over the years. Previous studies have compared transtibial technique with transportal techniques and reported better anatomic femoral tunnel placement with the latter^1-7^. In a study, Ahn *et al*^2^ found that the transportal technique allows for better anatomic femoral tunnel placement than the transtibial technique comparing the two techniques. Gadikota *et al*^4^ also found similar results in their study. Osti *et al*^7^ and Tompkins *et al*^1^ compared transtibial technique to AAM technique, and both found that AAM technique resulted in better placement. Shin *et al*^12^ discovered that the femoral tunnel was placed higher with the transtibial technique than with the low anteromedial technique in their study. They found no significant difference between deep and shallow positions.

Moon *et al*^13^ did a cadaveric study to show the influence of different anteromedial portals on the femoral tunnel orientation and reported that a mal-positioned AM portal results in abnormal tunnel orientation and significantly shorter tunnel length. They have also advised the use of AAM portal for better femoral tunnel placement. Our study also showed a statistically significant difference in deep to shallow position between the two techniques (AM technique superior to AL technique). Kim *et al*^14^ in their study showed the influence of knee flexion angles in creating the femoral tunnel. In our study, femoral tunnels were placed with knee in 120° flexion. Transportal technique has its own disadvantages particularly short tunnel length, medial femoral condyle cartilage damage, posterior blow out etc.

Recently, there has been emphasis on a horizontal angle of the femoral tunnel and a flat ACL graft for decreasing the risk of failure associated with a vertical graft. The use of a viewing anteromedial portal and drilling through an accessory anteromedial portal facilitated us to achieve this objective. Takeda *et al*^15^ reported that the AAM group's femoral tunnel angle is more horizontal than the TT group. Similarly, our study also revealed that the AM technique resulted in a more horizontal tunnel than the AL technique, which resulted in a more vertical tunnel, which was statistically significant.

Despite the study's main strength being CT-based measurement of femoral tunnel placements, there are a few limitations to this study. First and foremost, it is a retrospective study. Second, we have not examined the relationship between femoral tunnel malposition and clinical outcomes, highlighting the clinical significance. Third, we have not evaluated the complications associated with both techniques, such as medial femoral condyle damage, notch impingement, and so on, during tunnel placement.

## Conclusion

While performing ACLR, the anteromedial visualisation portal can facilitate accurate femoral tunnel placement compared to anterolateral visualisation portal. However, the clinical implications and its long-term outcomes needs to be studied.
